# Non-surgical treatment with XSHHD for ruptured lumbar disc herniation: a 3-year prospective observational study

**DOI:** 10.1186/s12891-020-03723-2

**Published:** 2020-10-19

**Authors:** Feng Dai, Yu Xiang Dai, Hong Jiang, Peng Fei Yu, Jin Tao Liu

**Affiliations:** Department of Orthopedics, Suzhou TCM Hospital affiliated to Nanjing University of Traditional Chinese Medicine, Suzhou, 215009 Jiangsu Province China

**Keywords:** Ruptured lumbar disc herniation, Non-surgical treatment, Xiao sui Hua He decoction, Traditional Chinese medicine, Resorption, Natural outcome

## Abstract

**Background:**

Lumbar disc herniation (LDH) is mainly caused by annular fiber disruption with a discrete leakage of nucleus pulposus pressing on a nerve, resulting in back pain and radiating pain. Most patients with LDH can be treated conservatively, but there are many different conservative treatments. Furthermore, most previous studies did not evaluate the long-term efficacy of these treatments and the prognosis. Therefore, an effective and safe therapeutic strategy is lacking for patients with LDH. In this study, we evaluated Xiao Sui Hua He decoction (XSHHD) in the treatment of LDH.

**Methods:**

This was a rigorous prospective observational 3-year follow-up study. We recruited 69 participants with ruptured lumbar disc herniation (RLDH) between February 2014 and February 2016. Patients took XSHHD orally twice a day for 6 months. The primary outcome measurements were visual analogue scale (VAS) pain score, Oswestry disability index (ODI) and straight leg raising test (SLRT). The secondary outcome measurements was nucleus pulposus protrusion volume on magnetic resonance imaging (MRI). Clinical outcomes were measured at baseline (Visit 1), and at 3, 6, 12, and 36 months (Visit 2, 3, 4, and 5, respectively)..

**Results:**

Sixty-three patients were followed-up for 3 years after treatment. SLRT and ODI after non-surgical treatment improved significantly compared with baseline (*P* < .001). There were no statistically significant differences at 6 months vs 36 months for SLRT and ODI. VAS scores (leg, back) after 3 years of treatment were statistically significantly different compared with baseline (*P* < .001; Z = − 6.93, − 6.637). The baseline protrusion volume was 2018.61 ± 601.16 mm^3^, and the volume decreased significantly to 996.51 ± 387.42 mm3 at 36 months (t = 12.863; *P* < .001). The volume of protrusion resorption rate (VPRR) at 36 months was 47.24 ± 23.99%, with significant resorption in 23 cases, partial resorption in 23 cases, no resorption in 15 cases, and increased volume in 2 cases.

**Conclusions:**

This study showed that non-surgical treatment with XSHHD was effective, and the study clarified the natural outcomes in LDH.

## Background

Lumbar disc herniation (LDH) is mainly caused by annular fibers disruption with a discrete leakage of nucleus pulposus pressing on a nerve, resulting in back pain, radiating pain in the lower extremities, and paresthesia as the main manifestations of the disease. Many clinical guidelines, including the Prognosis Research Trial and meta-analyses, acknowledge the effectiveness of conservative treatment for patients with LD H[1–4].

Herbal medicine is one of the most important parts of traditional Chinese medicine (TCM), with a history of thousands of years of use. Currently, herbal medicine is widely used in the treatment of LDH as an alternative and complementary therapy to Western medicine in China.

According to whether the posterior longitudinal ligament is ruptured, LDH can be divided into ruptured and non-ruptured types. Ruptured lumbar disc herniation (RLDH) is one of the most commonly accepted surgical indications due to the large-sized and dissociative protrusions and patients’ severe clinical symptoms. However, if RLDH can be controlled by drugs in the acute stage, resorption of the protrusions may occur after a period of time, and the clinical symptoms will also be alleviated. Even large nucleus pulposus protrusions can be absorbed spontaneously.

Some previous studies have evaluated non-surgical treatment (including herbal medicine) of RLDH,[5–7] but most have focused on short-term efficacy, with little mention of the long-term efficacy and prognosis of RLDH. We performed a rigorous prospective observational study designed to observe the near-term and mid-term efficacy and the natural long-term outcomes of non-surgical treatment of RLDH. We also evaluated the efficacy and safety of herbal medicine in RLDH.

## Materials and methods

### Study design

This study was a rigorous prospective observational 3-year follow-up study.

#### Study time and institution

We enrolled 66 patients with single-segment RLDH from the outpatient clinic of Suzhou TCM Hospital affiliated to Nanjing University of Traditional Chinese Medicine. The recruitment period was 24 months and extended between February 2014 and February 2016. The inclusion criteria were: (1) age 18–60 years; (2) symptom duration: ≤ 6 months; (3) visual analogue scale (VAS) score ≥ 4/10; (4) MRI data consistent with the symptoms of single-segment RLDH, with an interrupted posterior margin of the “black line” (posterior longitudinal ligament )[8]; (5) unilateral lower extremity radiating pain; and (6) signed informed consent. Exclusion criteria were: (1) concurrent lumbar vertebral dysplasia, spinal stenosis, lumbar spondylolisthesis, tumor, fracture, or infection; (2) pregnancy, breastfeeding, or pregnancy intent; (3) mental illness; (4) concurrent cardiac insufficiency, or liver or kidney dysfunction; (5) congenital abnormalities or a history of lumbar surgery; and (6) cauda equina syndrome with progressive neurological dysfunction, such as bladder and bowel dysfunction or saddle anesthesia.

### Interventions

Patients were instructed to take 120 mL Xiao Sui Hua He decoction (XSHHD) orally twice a day for 6 months. XSHHD was developed by Professor Jiang Hong, and was modified according to the Fang Ji Hang Qi decoction and Bu Yang Huan Wu decoction, which were the ancient Chinese prescriptions for thousands of years. XSHHD is widely used in the treatment of LDH in Suzhou TCM Hospital, and we performed a series of clinical and experimental studies to show that XSHHD is effective for LD H[6, 7, 9]. XSHHD is composed of the crude extracts of the herbs, *Astragalus mongholicus* (Huang Qi, 20 g), *Stephania tetrandra* (Fang Ji, 10 g), *Chinese angelica* (Dang Gui, 10 g), *Semen brassicae* (Bai Jie Zi,6 g), *Ligusticum wallichii* (Chuan Xiong, 15 g), *radix Clematidis* (Wei Lin Xian, 10 g), *fructus Chaenomeles lagenariae* (Mu Gua, 10 g), *white Atractylodes rhizome* (Bai Zhu, 10 g), *Pberetima* (Di Long, 10 g), and Aulastomum gulo (Shui Zhi, 6 g).

In the acute phase (within 2 weeks), patients were prescribed bed rest, and were permitted to use celecoxib temporarily, if necessary (Pfizer; 200 mg, twice a day). Rehabilitation training was increased during the remission stage, because this training can enhance the strength of the lumbar muscles, and prevent waist injury.

At baseline (Visit 1), all patients underwent MRI, and begin to take XSHHD. At 3, 6, and 12 months, patients visited the hospital again (Visit 2, 3, and 4, respectively) and underwent clinical examinations. At 36 months, the final visit (Visit 5) was performed as an outpatient consultation, and each patient underwent repeat MRI. During the follow-up, each patient visited the hospital within 3 days of the arranged time point. We made appointments for the patient at the outpatient clinic for the follow-up examinations, and recorded follow-up information and scores in a follow-up registration form. When serious adverse effects occurred, we provided immediate appropriate treatment, recorded the adverse effect, and stopped the medicine.

### Main outcome measures

At Visits 1 and 5, back pain and sciatica were evaluated using VAS ranging from 0 to 1 0[10].

At Visits 1–5, the symptoms of nerve root compression were measured by the straight leg raising test (SLRT),[11] which was performed with the patient in a supine position. The examiner gently raised the patient’s leg by flexing the hip with the knee in extension, and the test was considered positive when the patient experienced pain in the lower limb in the same distribution as the lower radicular nerve roots (usually L5 or S1). The examiners recorded the angle at which the patient’s leg was raised at the point of pain.

At Visits 1–5, spinal dysfunction was measured using the Oswestry disability index (ODI )[10]. The ODI improvement rate (OIR) at 36 months was calculated as follows: (Visit 1 ODI − Visit 5 ODI) / Visit 1 ODI) *100%.

### Secondary outcome measures

The secondary outcome measurement was nucleus pulposus protrusion volume on MRI. The final visit of volume of protrusion resorption rate (VPRR) was calculated as follows: (Visit 1 protrusion volume − Visit 5 protrusion volume) / Visit 1 protrusion volume *100%. Disc herniation resorption was classified as follows:

·Significant Resorption: VPRR ≥60%
·Partial Resorption: VPRR ≥30% to < 60%
·None Resorption: VPRR ≥ − 20% to < 30%
·Increased: VPRR < − 20%.

**Fig. 1:**
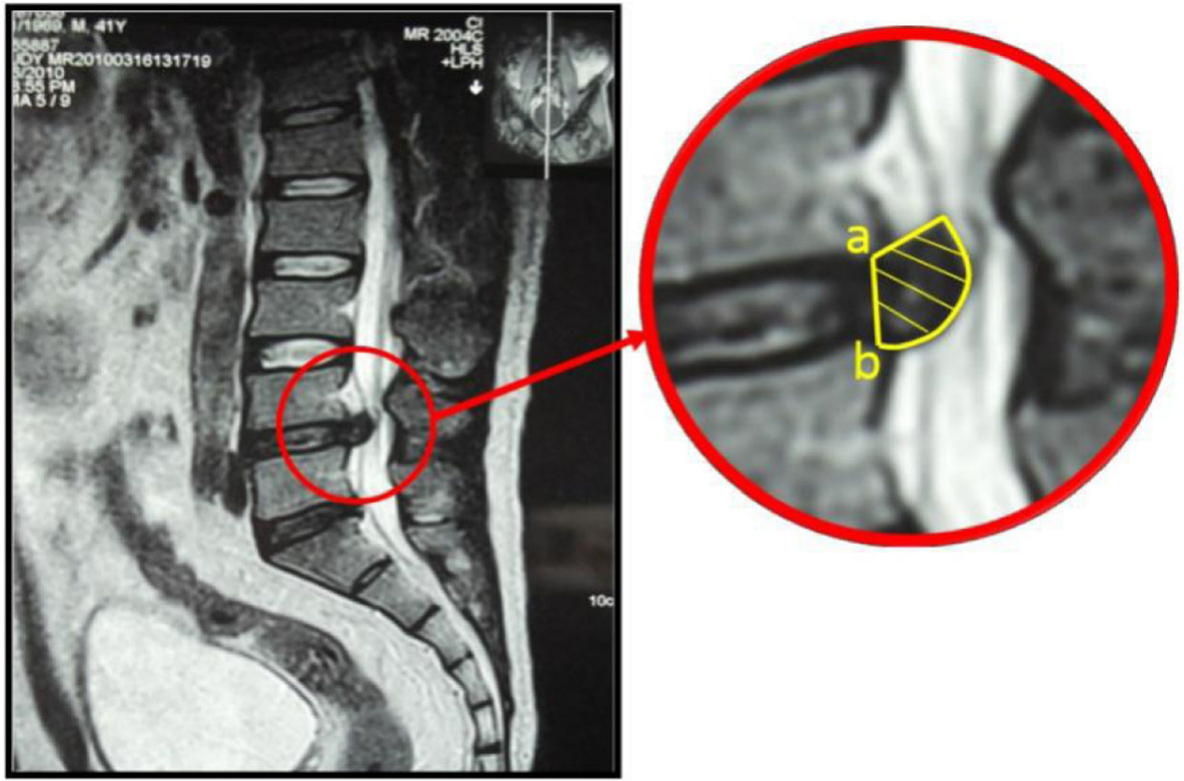
Method of measuring the protrusion area: On T2WI sagittal images, the upper edge of the vertebral bodies and the vertebral body (**a**) after the edge (**b**) on the attachment marked the boundaries, and section in yellow indicates the protrusion

Protrusion volume measurement was performed using a Siemens 1.5 T superconducting MRI unit (magnetoionic intensity: 0.35 Tes/a; spin-echo sequence; 11 layers scanned at the sagittal position; interlayer spacing: 1.25 mm; layer thickness: 5 mm). The image data were processed using picture archiving and communication systems (PACS). The volume of the protrusion was calculated by the method described by Auti o[12]. The T2WI sagittal image was taken from the PACS system. The posterior inferior margin of the upper vertebral body and the posterior superior margin of the lower vertebral body were used as the inner boundary; the posterior margin of the protrusion acted as the outer boundary; and the area of the protrusion was then calculated using software (Fig. 1). The volume of the protrusion (VP, mm^3^) was calculated by the equation:

$$ VP=\left( IS+ LT\right)\times \sum \limits_i^nA{P}_i $$

Where IS represents interlayer spacing, LT represents layer thickness, AP_i_ represents the area of the protrusions of layer *i*, and the total layer number is n.

### Statistical analysis

Statistical analyses were performed using SPSS Statistics software (version 23.0). Continuous data are presented as mean ± standard deviation (SD), and categorical data are presented as frequencies. The comparison of SLRT and ODI was performed by the Friedman M test of the non-parametric test. Comparisons of protrusions volumes were performed by the paired t-test. VAS (leg, back) comparisons were performed by Wilcoxon’s nonparametric test, and Spearman’s correlation analysis was used to compare OIR and VPRR. *P* < .05 was considered statistically significant.

### Ethical issues

This study was conducted in accordance with the Declaration of Helsinki (WMA) and the International Ethical Guidelines for Biomedical Research involving human subjects (CIOMS). This was study was reviewed and approved by the Ethics Committee of Suzhou Hospital of Traditional Chinese Medicine, and the approval number was 2017-LYP-013.

## Results

### Clinical results

**Fig. 2:**
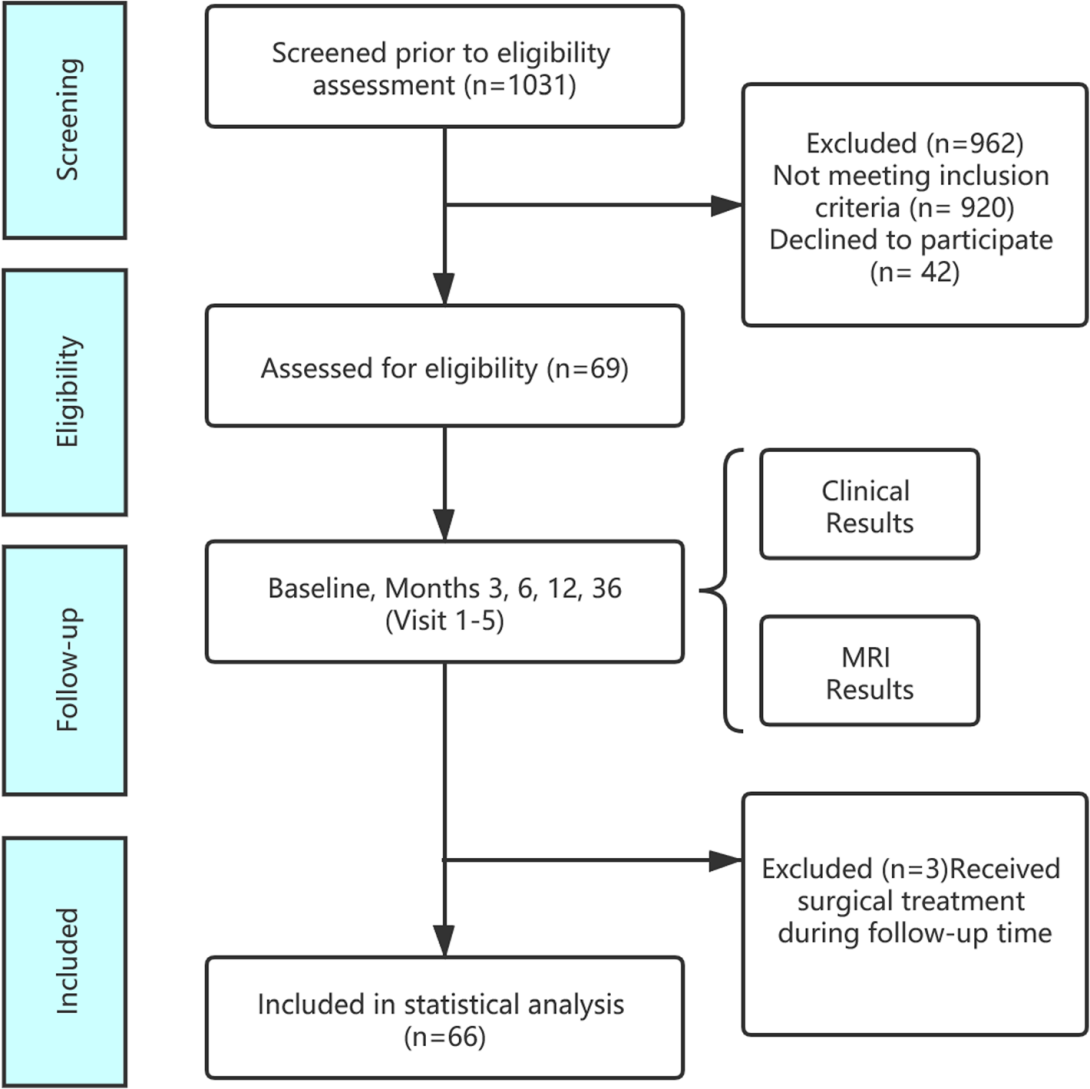
Flow diagram

Of 1031 patients screened, 69 participants were recruited, and 66 concluded 6 months’ treatment and follow-up. Three patients received surgical treatment, among whom 2 patients had progressive pain seriously affecting work and life during the treatment, and 1 patient developed cauda equina syndrome during the treatment. The 66 patients constituted 46 males and 20 females, aged 18 to 58 years, with an average of 36.27 ± 9.97 years. Lesions were located at L3/4 in 4 cases, L4/5 in 27 cases, and L5/S1 in 35 cases. Patients underwent bed rest from 1 to 3.5 weeks, with an average of 2.19 ± 0.54 weeks. Patients took XSHHD from 8 weeks to 6 months, with an average duration of 16.86 ± 5.55 weeks. The study flow diagram is presented in Fig. 2.

SLRT and ODI values after non-surgical treatment (Visits 2, 3, 4, 5), improved significantly after treatment compared with baseline, and the differences were statistically significant (*P* < .001). VAS (leg, back) scores 3 years after treatment were statistically significanly different compared with baseline values (P < .001; Z = − 6.93, − 6.637) (Table 1).

**Table 1:** Comparisons between SLRT, VAS, ODI, and the ODI improvement rate (OIR), in degrees (°), score, percentage (%), and score, respectively.

Time	Baseline	3 months	6 months	12 months	36 months	*P* value
SLRT (mean ± S.D.)	30.16 ± 15.79	59.05 ± 11.74	68.10 ± 10.45	74.52 ± 10.03	69.76 ± 9.69	*P* < 0.001
ODI (mean ± S.D.)	40.19 ± 6.59	13.76 ± 4.73	8.83 ± 3.27	6.35 ± 2.88	9.95 ± 4.95	*P* < 0.001
OIR (mean ± S.D.)	–	–	–	–	75.05 ± 12.66	–
VAS-Leg (mean ± S.D.)	7.31 ± 1.02	–	–	–	1.45 ± 1.26	*P* < 0.001
VAS-Back (mean ± S.D.)	4.77 ± 0.94	–	–	–	1.97 ± 1.42	*P* < 0.001

*S.D.* standard deviation. The number of cases was 63

#### Clinical efficacy

There was no statistically significant difference between Visit 5 and 3 for ODI (q = −.183; *P* = .517). However, a statistically significant difference was found in pair-wise comparisons at the other visit points (*P* < .001),indicating that non-surgical treatment with XSHHD was effective. The curative effect continued to improve from 3 months to 12 months, and thereafter, the curative effect gradually decreased. However, the overall clinical efficacy at 36 months was equivalent to that at 6 months.

#### Clinical function

There was no statistically significant difference between Visit 5 and 3 for SLRT (q = − 214; *P* = .447). However, a statistically significant difference was found in pair-wise comparisons at the other visit points (*P* < .01), indicating that clinical function steadily improved from 3 months to 6 months, with the best function at 12 months. With longer follow-up function decreased, but was still significantly improved compared with baseline, which was consistent with the clinical efficacy.

### MRI results

**Fig. 3:**
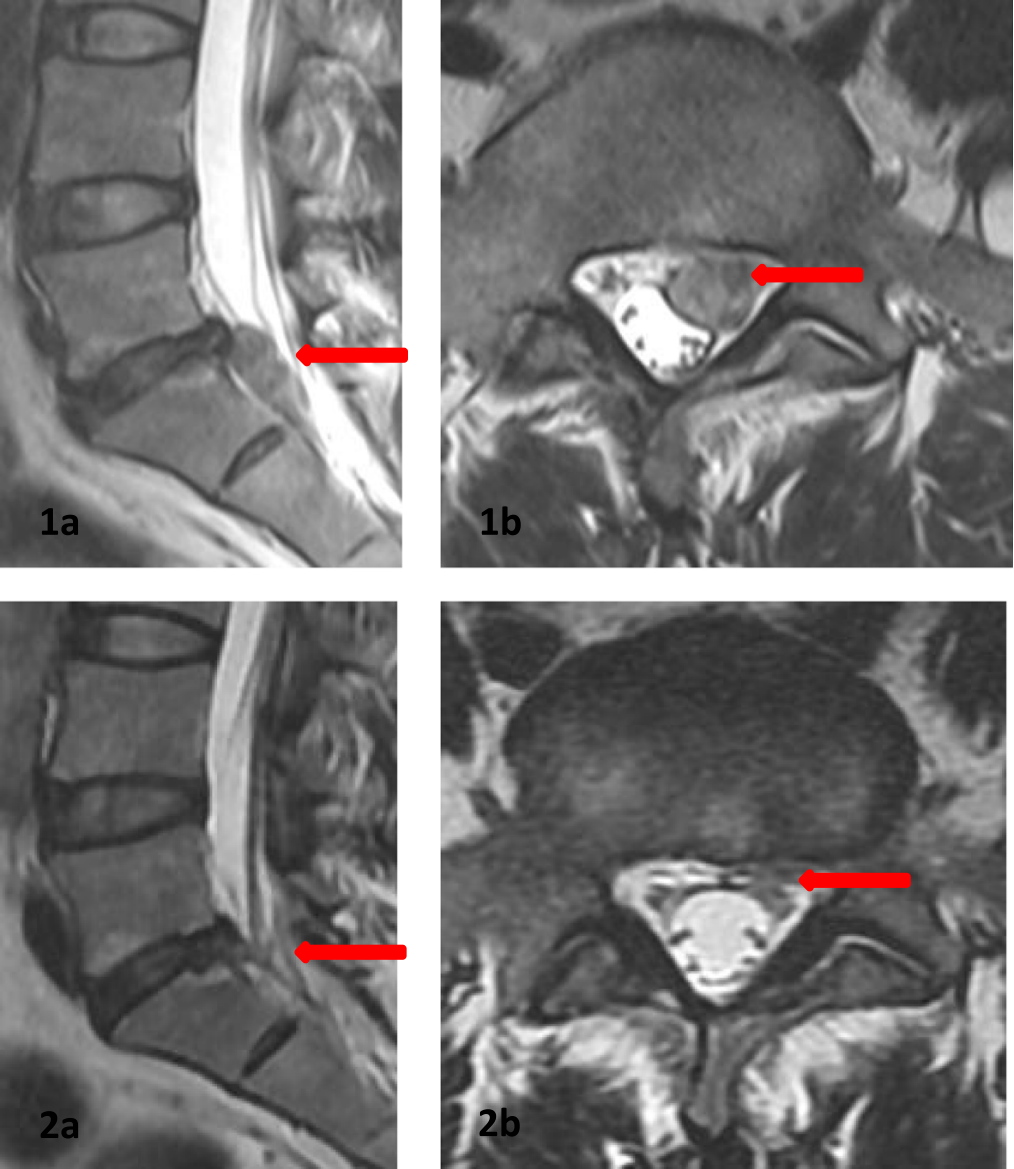
Male patient, 41 years old, with low back pain with referred left leg pain for 3 weeks. MRI indicates that a huge ruptured lumbar disc herniation on the left at level L5/S1. 1a and b: The baseline volume of the protrusion is 3307.0 mm^3^. SLRT: left 15^o^, right 60^o^, JOA score: 49. 2a and b: The volume of the protrusion was 486.46 mm^3^ at the last follow-up. SLRT: left 70^o^, right 70^o^, JOA score: 5

Comparisons of the MRI findings in the 63 patients between Visit 5 and baseline are shown in Table 2. The baseline protrusion volume was 2018.61 ± 601.16 mm^3^, and the volume decreased significantly to 996.51 ± 387.42 mm^3^ at 36 months(t = 12.863; *P* < .001). The VPRR at 36 months was 47.24 ± 23.99%, with significant resorption in 23 cases, partial resorption in 23 cases, no resorption in 15 cases, and increased volume in 2 cases (Table 2, Figs. 2 and 3).

**Table 2:** Comparisons of the MRI findings between baseline and 36 months

Time	PV	VPRR	Variation of the protrusion resorption (cases)			
			Significant	Partial	None	Increased
baseline	2018.61 (601.16)	–	–	–	–	–
36 Months	996.51 (387.42)	47.24 (23.99)	23	23	15	2

*PV* Protrusion volume (mm^3^); *VPRR* volume of protrusion resorption rate(%),

T statistics = 12.863, *p*-value < .001

The number of cases is 63

### Correlation analysis

After 36 months’ treatment, the Spearman rank correlation analysis was performed on the 63 patients with VPRR and OIR. It was found that there was a positive correlation between the two (r = 0.682, *P* < .001), showing that the higher the improvement rate of ODI in patients was, the better the protrusion resorption rate (RR).

## Discussion

In this study, the VPRR at 36 months was 47.24 ± 23.99%, with significant resorption in 23 cases, partial resorption in 23 cases, no resorption in 15 cases, and increased volume in 2 cases. Resorption is common in RLDH, and this has great significance for the non-surgical treatment of the disease. In this study, the curative effects steadily improved from 3 months to 12 months after non-surgical treatment with XSHHD. With longer follow-up time, the effect decreased, but significant improvement remained compared with baseline. At the last follow-up, five patients had experienced recurrence and pain in the back and lower extremities, but all patients refused surgery and were willing to accept non-surgical treatment again. Furthermore, after 3 years’ treatment, most patients reported no pain and had obvious improvement in their symptoms. These findings indicate that non-surgical treatment of RLDH with XSHHD is effective. Patients took XSHHD for less than 6 months, and with the complete metabolism and excretion from the body, the effect of XSHHD will be lost after that time. In this study, we evaluated the mid- and long-term follow-up of the disease (> 6 months after treatment), and the findings are considered the natural outcome of RLDH. Long-term, there is a certain recurrence rate with RLDH. In this study, after 1 year of treatment, patients’ symptoms were slightly worse than before, but their overall condition was satisfactory, with no major adverse effects.

“Resorption” refers to the protrusion reduction or even disappearance without surgical intervention in LDH patients. Since Guinto et al. first discovered protrusion resorption on CT in 1984,[13] this phenomenon has received greater attention from clinicians. The mechanisms of resorption in RLDH mainly include: 1) Autoimmune response: After breaking through the posterior longitudinal ligament, the protrusion directly contacts the blood supply and is recognized as an antigen by the immune system, thereby causing immune lysi s[14, 15]. 2) Vascularization: The protruding tissue directly enters the epidural space, stimulating the growth of new blood vessels and promoting the infiltration of macrophages as well as phagocytosi s[16, 17]. 3) Inflammatory reactions: Inflammatory cell infiltration secondary to the release of various inflammatory mediators and mononuclear macrophages promotes the resorption of the protrusion s[18, 19]. 4) Matrix degradation and apoptosis: The activity of matrix metalloproteinases (MMPs) and related cytokines (e.g., TNF, IL) increases thus, tissue degradation and apoptosis are promote d[20, 21]. 5) Tissue dehydration and hematoma absorptio n[22, 23].

Currently, RLDH is generally included in the clinical indications for surgery. However, non-surgical treatment is a definite option for patients without cauda equina syndrome whose life and work are not seriously affected. In a 2-year prospective randomized controlled trial conducted by Atlas et al.,[24] 924 patients achieved satisfactory results with non-surgical treatment. David et al .[25] found that the clinical symptoms of most non-surgical patients improved significantly after more than 5 years of follow-up. After 8 years of follow-up, Lurie et al .[26] also found that both surgical (501 cases) and non-surgical (743 cases) treatment of LDH provided satisfactory results. Some specialists have pointed out that although the short-term efficacy of surgical treatment for LDH is better than non-surgical treatment, there is no statistical difference between the two approaches after 52 week s[27].

Many studies have shown that TCM can achieve good results in the treatment of LD H[28, 29]. In a 5-year follow-up of non-surgical treatment of LDH using herbal medicine, Shin et al .[30] found that patients’ symptoms improved significantly, and their satisfaction rate was high. Professor Hong’s XSHHD used in this study was prescribed and modified according to the ancient Chinese prescriptions. Modern pharmacological studies have shown that Huang Qi can improve the immune response, promote the proliferation of Schwann cells, accelerate axonal growth, and improve nerve regeneration repair functio n[31, 32]. Dang Gui can expand blood vessels, and promote blood circulation and the growth of new blood vessels in prominent tissue s[33]. Fang Ji is believed to have anti-inflammatory and analgesic effects, and to be involved in dehydration and relieving swelling, as well as muscle relaxation and regulating autoimmunity. Mu Gua and Wei Lin Xian decrease inflammatory reactions, promote regression of nerve root edema, and reduce connective tissue hyperplasia and adhesion formation. These drugs work together to relieve the clinical symptoms of LDH and promote resorption through some of the mechanisms described above.

Most of the patients in this study were satisfied with the treatment results, and no adverse effects or complications occurred during the study period. Our results showed that XSHHD is safe, and that non-surgical treatment with XSHHD is a feasible treatments for RLDH.

## Conclusions

As a rigorous prospective observational 3-year follow-up study, all patients in this study were followed–up for clinical efficacy (ODI, VAS) and functional recovery (SLRT). Additionally, MRI examinations were performed at baseline and at the last follow-up. Our study proved that non-surgical treatment with XSHHD is effective, and we clarified the natural outcome of the RLDH. Owing to the small sample size and lack of a randomized control group in this study, it is difficult to compare the current treatment with other treatments. This study has limitations, and large-sample prospective randomized case–control studies are needed to further evaluate the efficacy of treatment with XSHHD.

## Data Availability

The datasets used and/or analyzed during the current study are available from the corresponding author on reasonable request.

## Abbreviations

XSHHD: Xiao Sui Hua He decoction
TCM: Traditional Chinese medicine
LDH: Lumbar disc herniation
RLDH: Ruptured lumbar disc herniation
VAS: Visual analogue scale
ODI: Oswestry disability index
SLRT: Straight leg raising test
OIR: ODI improvement rate
VPRR: Volume of protrusion resorption rate
MRI: Magnetic resonance imaging
PACS: Picture archiving and communication systems
CRF: Case report form
SD: Standard deviation
MMPs: Matrix metalloproteinases
TNF: Tumor necrosis factor
IL: Interleukin
